# Anti-α-1,4-D-polygalacturonic acid antibodies as a new biomarker for juvenile idiopathic arthritis

**DOI:** 10.1007/s10067-024-07061-9

**Published:** 2024-07-13

**Authors:** Jiqian Huang, Zhijing Wu, Wei Quan, Xiaohua Ye, Xiaolong Dai, Jiangtao Luo, Xiao Han, Xiaozhong Li, Wenjie Zheng

**Affiliations:** 1 Department of Pediatric Rheumatology, The Second Affiliated Hospital and Yuying Children’s Hospital of Wenzhou Medical University, Zhejiang Province, Wenzhou, 325027 China; 2 Rheumatology and Immunology Department, Children’s Hospital of Nanjing Medical University, Nanjing, China; 3 Department of Physics, University of Cambridge, Cambridge, UK; 4 Department of Nephrology and Immunology, Children’s Hospital of Soochow University, Suzhou, Jiangsu Province 215003 China

**Keywords:** α-1,4-D-polygalacturonic acid antibody, Biomarker, Disease activity, Juvenile idiopathic arthritis

## Abstract

**Objective:**

Diagnosing juvenile idiopathic arthritis (JIA) is challenging. Our study aimed to investigate the clinical significance of anti-α-1,4-D-polygalacturonic acid (PGA) antibodies in JIA, focusing on their role in diagnosis and assessing disease activity.

**Methods:**

In this prospective case–control study, we examined variations in serum levels of PGA-IgA and PGA-IgG among children with different types of JIA and healthy controls. Serum PGA-IgA and PGA-IgG levels were assessed concurrently in children with active and inactive JIA.

**Results:**

This study included 126 patients diagnosed with JIA, 13 neonates, and 76 healthy children. Serum PGA-IgA and PGA-IgG levels were assessed, which revealed significant differences in PGA-IgA levels between various JIA subtypes and controls. An analysis of PGA-IgA levels in various JIA states revealed a statistically significant difference. Receiver operating characteristic (ROC) analysis demonstrated the robust predictive capability of PGA-IgA, with an AUC of 0.879 (*p* < 0.001), along with a specificity of 0.842 and sensitivity of 0.848.

**Conclusion:**

Increased levels of anti-PGA antibodies, particularly PGA-IgA, were significantly associated with JIA. PGA-IgA may serve as a sensitive biomarker for disease activity in JIA and could potentially aid in the diagnosis of JIA.

**Table:** 

**Key Points** • *This study found a significant correlation between blood levels of PGA-IgA and juvenile idiopathic arthritis (JIA), which may provide valuable diagnostic insights.* • *PGA-IgA shows potential as a sensitive biomarker for the assessment of disease activity in JIA patients, helping to determine disease activity.*

## Background

Juvenile idiopathic arthritis (JIA) encompasses a spectrum of chronic arthritic conditions that manifest in childhood. Although it is commonly perceived as a singular entity, JIA is associated with a diverse range of inflammatory arthropathies. According to the 2001 classification criteria established by the International League of Associations for Rheumatology (ILAR) [1], JIA is categorized into seven distinct subtypes, including systemic JIA (sJIA), rheumatoid factor (RF)-negative polyarticular JIA (pJIA), RF-positive pJIA, oligoarticular JIA (oJIA), enthesitis-related arthritis (ERA), psoriatic JIA, and undifferentiated JIA. JIA presents a wide arrange of clinical manifestations, highlighting its marked heterogeneity. In certain instances, the onset of JIA may be insidious, leading to misdiagnosis and missed opportunities for timely intervention. Such delays can result in protracted disease courses and unfavorable prognoses.

However, the precise etiology and pathogenic mechanisms of JIA remain unclear. Interactions between genetic factors, immune mechanisms, and environmental exposure may play a role in the pathogenesis of most affected children, and the pathogenicity of autoantibodies has been confirmed. Currently, the medical community unanimously recognizes the high diagnostic and prognostic value of RF and anti-cyclic citrullinated peptide (anti-CCP) antibodies [2]. In children with JIA, the presence of RF and anti-CCP antibodies was associated with a higher likelihood of erosive joint damage. However, unlike adults, the positivity rate of these antibodies in JIA is < 20% and primarily occurs in the polyarticular subtype [3, 4]. Therefore, considering additional autoantibodies is crucial to enhance early diagnosis and improve disease prognosis.

In patients with rheumatoid arthritis (RA), IgG fragments that bind to various proteins and peptides were isolated and purified from the synovial membrane and cartilage immune complexes in 2009 [5]. Subsequently, elevated levels of diverse antiglycan antibodies have been identified in autoimmune diseases such as systemic lupus erythematosus (SLE), antiphospholipid antibody syndrome, and IgA nephropathy. Pectin is a polysaccharide macromolecule that is widely used as an ingredient in the food industry whose main component is alpha-1,4-polygalacturonic acid (PGA). Fermented dairy products commonly contain food thickeners, with starch and pectin being the most frequently added [6, 7]. This raises intriguing questions about the potential impact on the immune system arising from the routine consumption of such products. Research has indicated that serum levels of PGA-IgA and PGA-IgG in patients were significantly higher than those in healthy controls [8]. Immunohistochemical staining results revealed that the PGA-Abs were selectively positively stained in the synovial membrane and chondrocytes of the cartilage but not in any other tissues, including the spleen, lung, kidney, and liver. This suggests that diet-derived PGA antigens and anti-PGA antibodies may play a role in RA pathogenesis [8].

Currently, research on the correlation between the PGA and JIA is lacking. This study aimed to explore the relationship between anti-PGA antibodies and the different subtypes of JIA. This study will further investigate whether anti-PGA antibodies can serve as potential biomarkers for JIA disease activity and complementary markers for JIA diagnosis.

## Methods

### Patient and sample

This prospective case–control study enrolled pediatric patients from the Second Affiliated Hospital of Wenzhou Medical University and the Children’s Hospital of Soochow University. All children with JIA met the classification criteria revised by the ILAR in 2001 [9]. Children with infectious or malignant diseases were excluded. The JIA group was further stratified into two subgroups: active (Group A) and inactive (Group B) states. The active state was defined as a child with JIA exhibiting active disease without treatment or relapse after treatment. The inactive state was characterized as being inactive for more than 6 months. Inactive state met the Wallace criteria [10], which were defined as (1) no joints with active arthritis, (2) no fever, rash, serositis, splenomegaly, or generalized lymphadenopathy attributable to JIA, (3) no active uveitis to be defined, (4) normal erythrocyte sedimentation rate (ESR) and C-reactive protein (CRP) levels, and (5) a physician’s global assessment of disease activity score of the best possible on the scale used. Normal controls consisted of healthy children who underwent physical examinations at the Child Health Clinic. Neonates were included to establish baseline serum antibody levels. Information about demographic and clinical features and laboratory data were recorded and analyzed, including age, sex, number of swollen joints, number of tender joints, ESR, CRP, white blood cell count (WBC), serum levels of immunoglobulins (IgG, IgM, and IgA), complement (C3, C4), rheumatoid factor (RF), anti-citrullinated peptide (anti-CCP) antibodies, Juvenile Arthritis Disease Activity Score 27-joint reduced count (JADAS27) or systemic-onset JADAS27 (sJADAS27).

This study was approved by the Ethics Committee of the Second Affiliated Hospital of Wenzhou Medical University (2021-K-258–02) and Children’s Hospital of Soochow University (2020CS083) and written informed consent was obtained from the patients’ parents. Each participant provided a peripheral venous blood sample (3–5 ml). These samples were promptly stored at room temperature, and serum separation was completed within 4 h of collection. Serum samples that could not be analyzed within 8 h of collection were stored at − 80 °C prior to analysis, with strict avoidance of repeated freeze–thaw cycles.

### Serum PGA-IgA and PGA-IgG determination

NuncTM 96-well plates (Thermo Fisher Scientific, Roskilde, Denmark) were coated with 50 µg/ml PGA at 4 °C overnight. Subsequently, the plates underwent blocking with 3% bovine serum albumin (BSA; Solarbio, Beijing, China) at 4 °C overnight. Serum samples, diluted 1:200 in 3% BSA, were then added to the poly-L-lysine-coated wells of the 96-well plate and incubated at 37 °C for 2 h. After three washes with 0.05% Tween-20, 100 µL of either horseradish peroxidase (HRP)-conjugated IgA or HRP-conjugated IgG (Santa Cruz, CA, USA; both diluted 1:4000 with phosphate-buffered saline [PBS; pH 7.4]) was introduced to each well. Plates were covered and underwent a 1-h incubation at 37 °C before four washes with 0.05% Tween-20. The signal development occurred for 30 min at 37 °C using a chromogenic reagent kit (Yingke Xinchuang Technology, Nanjing, China). Afterward, a stop solution (Yingke Xinchuang Technology) was added according to the manufacturer’s instructions. Signal evaluation was conducted using a microplate reader (Bio-Rad, Hercules, CA, USA) to obtain optical density (OD) values at 450 nm.

### Statistical analyses

Data analyses were performed using the SPSS software (version 21.0; IBM, Armonk, NY, USA). Normally distributed continuous data are presented as the mean ± SD. The Dunnett *t*-test was used for pairwise comparisons between multiple patient groups and one control group. Between-group comparisons were performed using a *t*-test. Spearman’s rank correlation test was used to assess the relationships between two variables. The correlation was considered significant at *p* < 0.05. Binary logistic regression was used to assess the association between disease state, inflammatory factors, and elevated PGA-IgA levels. The sensitivity and specificity of PGA-IgA in detecting JIA and its ability to determine the indications for PGA-IgA were evaluated using a receiver operating characteristic (ROC) curve. A *p* value < 0.05 was considered to be statistically significant.

## Results

### Clinical characteristics of patients with *JIA*

This study included 126 patients diagnosed with JIA, comprising 71 males and 55 females, with a mean age of 10.57 ± 3.92 years (range, 2–17 years). Among them, 49 were in an active state, including 21 cases of enthesitis-related arthritis (ERA), 5 cases of sJIA, 10 cases of pJIA, and 13 cases of oJIA. Additionally, 77 children were in an inactive state: 27 with ERA, 20 with sJIA, 14 with pJIA, and 16 with oJIA. Other study groups included 13 neonates (6 males and 7 females) with a mean age of 7.85 ± 6.12 days (range, 2–20 days) and 76 healthy children (39 males and 37 females) with a mean age of 8.16 ± 3.37 years (range, 2–14 years). The clinical characteristics of patients are presented in Table 1, and in group A, the mean number of painful joints was 2.46 ± 1.84, while the mean of swollen joints was 1.27 ± 1.59. The JADAS27 score was recorded as 9.61 ± 4.31, and the sJADAS27 score was 13.46 ± 6.97. Conversely, these indicators were within the normal range in group B.

**Table 1:** The clinical characters of patients with JIA

Index	Group A (*n* = 49)	Group B (*n* = 77)
Male/female	25/24	46/31
Age	10.31 ± 3.92	10.74 ± 3.93
CRP (g/L)	24.65 ± 32.26	1.32 ± 2.64
WBC (*10^9^/L)	9.12 ± 3.95	6.52 ± 1.67
ESR (mm/h)	29.63 ± 24.63	6.45 ± 4.99
D-dimer (µg/ml)	1.16 ± 1.85	0.25 ± 0.32
RF (IU/L)	24.70 ± 81.38	11.14 ± 41.69
Anti-CCP (IU/L)	14.73 ± 48.21	33.40 ± 129.08
IgG (g/L)	13.37 ± 3.30	10.76 ± 3.16
IgA (g/L)	2.53 ± 1.06	1.28 ± 0.72
IgM (g/L)	1.56 ± 0.77	1.22 ± 0.36
C3 (g/L)	1.23 ± 0.25	0.89 ± 0.23
C4 (g/L)	0.30 ± 0.10	0.17 ± 0.07

*CRP* C-reactive protein, *WBC* white blood cells, *ESR* erythrocyte sedimentation rate, *RF* rheumatoid factor, *Anti-CCP* anti-cyclic citrullinated peptide, *IgG* immunoglobulin G, *IgA* immunoglobulin A, *IgM* immunoglobulin M, *C3* complement 3, *C4* Complement 4.

### Correlation between PGA-Ab level and different types of *JIA*

**Fig. 1:**
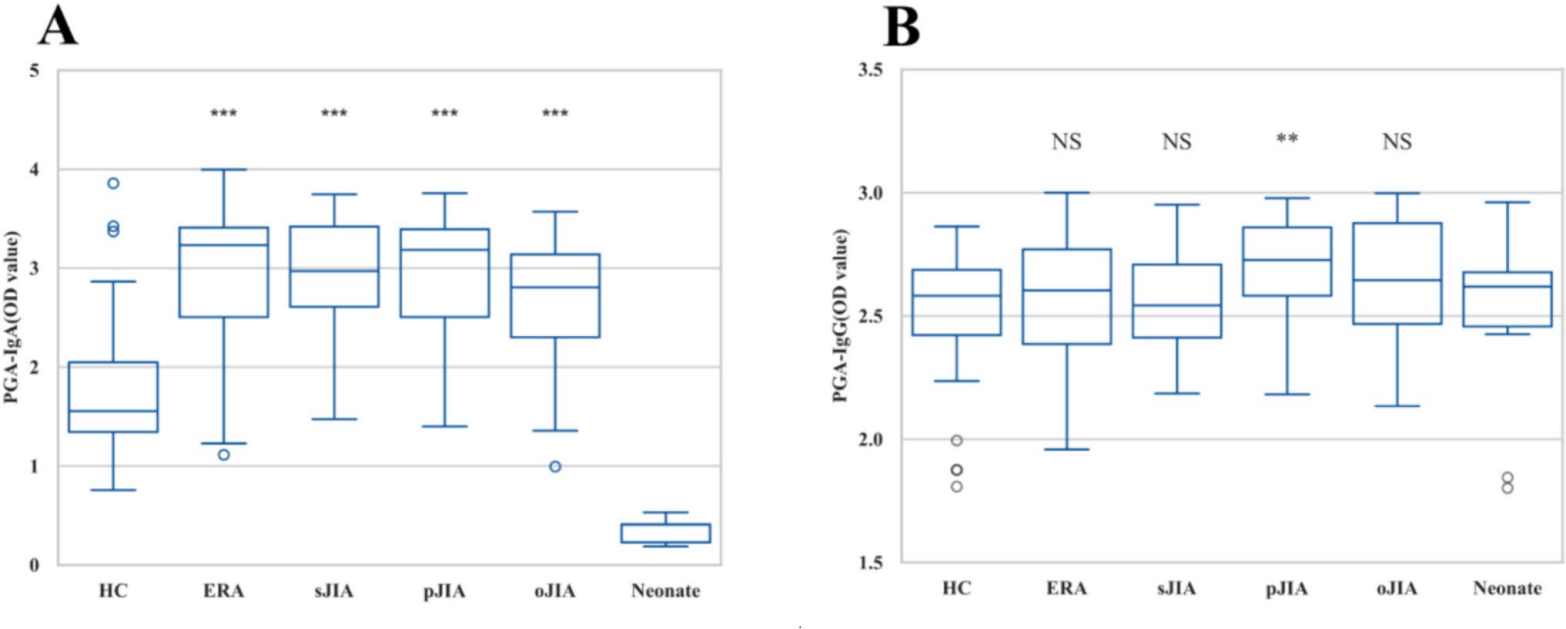
Serum PGA-IgA and PGA-IgG levels between patients with JIA, neonates, and healthy children. **A** Differences in serum PGA-IgA levels between various JIA subtypes and the control group. **B** Differences in serum PGA-IgG levels between various JIA subtypes and the control group. OD, optical density at 450 nm; NS* p* > 0.05, **p < 0.01, ***p < 0.0001

To analyze the correlation between the prevalence of PGA-Abs and different types of JIA, serum samples from patients with JIA (*n* = 126) and healthy children (*n* = 76) were individually tested for PGA-specific IgA and IgG levels (Table 2). The PGA-IgA levels in children with ERA, sJIA, pJIA, or oJIA were significantly higher than those in healthy children. However, only PGA-IgG levels in pJIA were significantly higher than those in healthy children (Fig. 1).

**Table 2:** Levels of serum PGA-IgA and PGA-IgG between patients with JIA and different control groups

Study group	Patient, *n* (%)	Anti-PGA molecules	
		PGA-IgA	PGA-IgG
JIA	126	2.87 ± 0.67	2.62 ± 0.23
ERA	48(38%)	2.97 ± 0.67***	2.57 ± 0.25
sJIA	25(20%)	2.82 ± 0.61***	2.47 ± 0.21
pJIA	24(19%)	2.88 ± 0.61***	2.62 ± 0.19**
oJIA	29(23%)	2.55 ± 0.69***	2.58 ± 0.23
Healthy children	76	1.75 ± 0.61	2.54 ± 0.22
Neonates	13	0.35 ± 0.13	2.33 ± 0.32

*PGA* a-1,4-D-polygalacturonic acid, *Ig* immunoglobulin, *JIA* juvenile idiopathic arthritis, *ERA* enthesitis-related arthritis, *sJIA* systemic juvenile idiopathic arthritis, *pJIA* polyarticular juvenile idiopathic arthritis, *oJIA* oligoarticular juvenile idiopathic arthritis; **p < 0.01, ***p < 0.0001.

It should be noted that the mean age of the HC group was younger than that of the JIA group. So we stratified JIA and HC groups into two subgroups based on age, then compared the differences in PGA levels between the two different age subgroups. Our analysis revealed no statistically significant differences in PGA-IgA levels between the age-stratified subgroups within either the JIA group or the HC group (*p* > 0.05) (Table 3).

**Table 3:** Levels of serum PGA-IgA in different age groups

Study group	Age (year)	N	PGA-IgA	*p* value
JIA	< 10.57	54	2.78 ± 0.71	0.1647
	> 10.57	72	2.95 ± 0.62	
HC	< 8.16	42	1.67 ± 0.56	0.2517
	> 8.16	34	1.84 ± 0.65	

*PGA* a-1,4-D-polygalacturonic acid, *Ig* immunoglobulin, *JIA* juvenile idiopathic arthritis.

**Fig. 2:**
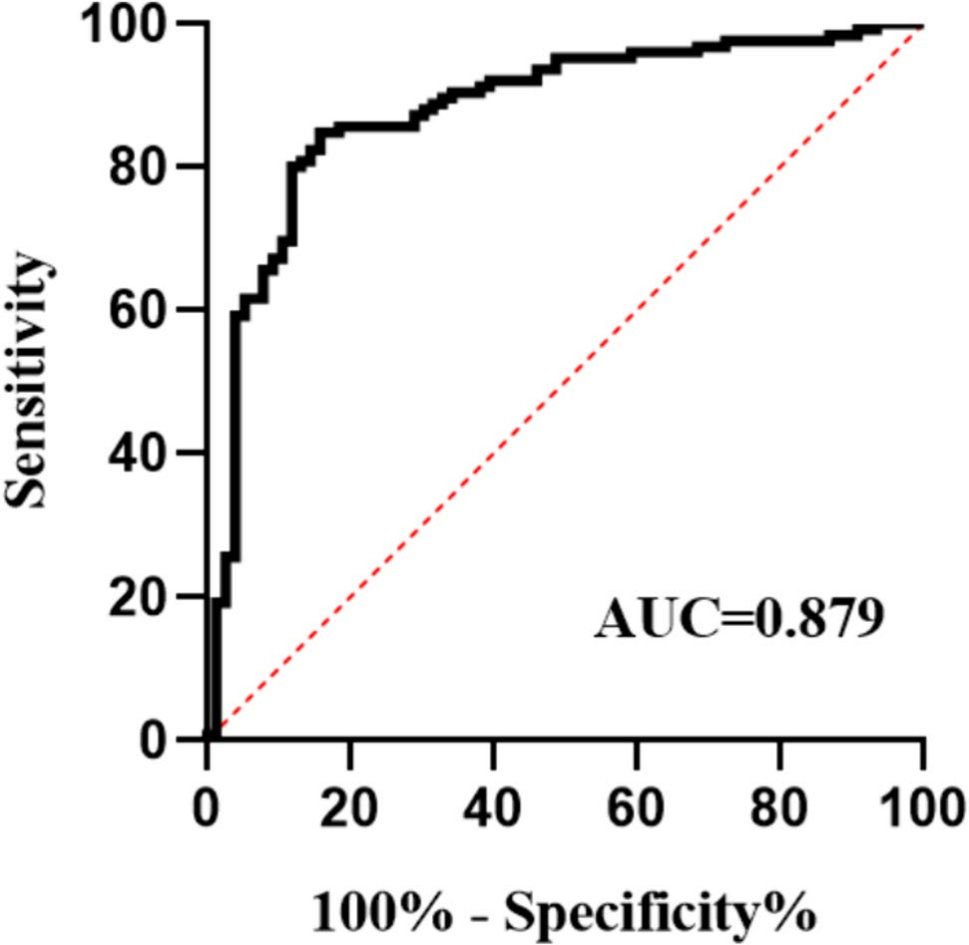
The AUC values are given in the figure

Receiver operating characteristic (ROC) curve analysis was performed using PGA-IgA as a test variable. The ROC analysis indicated that PGA-IgA possessed substantial predictive capability, with an area under the curve (AUC) of 0.879 (95% confidence interval, 0.828–0.931), which was statistically significant (*p* < 0.0001). Furthermore, the analysis yielded a specificity of 0.842, sensitivity of 0.848, Yoden index of 0.690, and cutoff value of 2.223 (Fig. 2).

### Correlation between PGA-IgA level and *JIA* disease activity

**Fig. 3:**
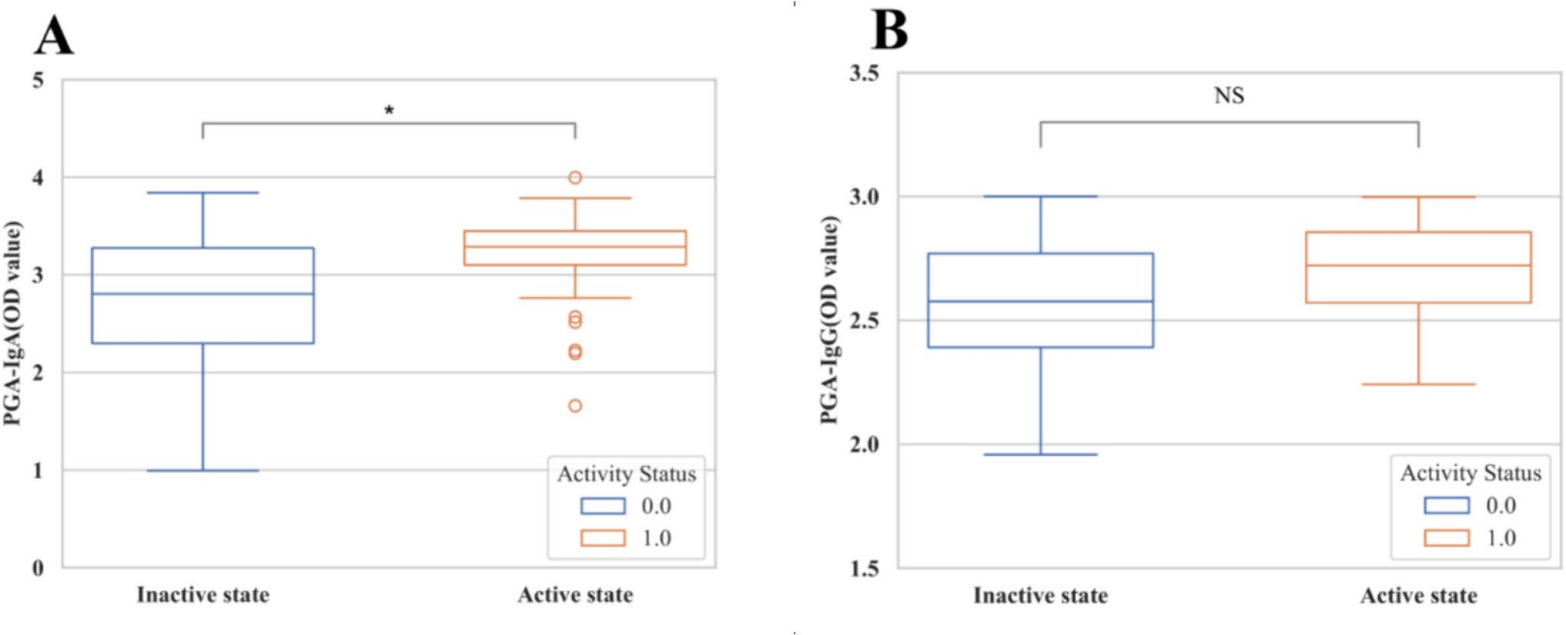
Serum PGA-IgA and PGA-IgG levels between active and inactive states. **A** Differences in serum PGA-IgA levels between active and inactive states. **B** Differences in serum PGA-IgG levels between active and inactive states. NS* p* > 0.05, *p < 0.05

To further analyze the correlation between PGA-IgA levels and JIA disease activity, serum samples from 44 patients with active JIA and 77 with inactive JIA (*n* = 77) were individually tested for PGA-specific IgA and IgG (Fig. 3). The average levels of PGA-IgA in the active state (3.21 ± 0.43) were significantly higher than those in the inactive state (2.67 ± 0.70) (*p* < 0.05), and the average levels of PGA-IgA in the inactive state (2.67 ± 0.70) were significantly higher than those in the HC group (1.75 ± 0.61) (*p* < 0.0001). However, no significant difference in PGA-IgG levels was observed between the active and inactive states.

**Fig. 4:**
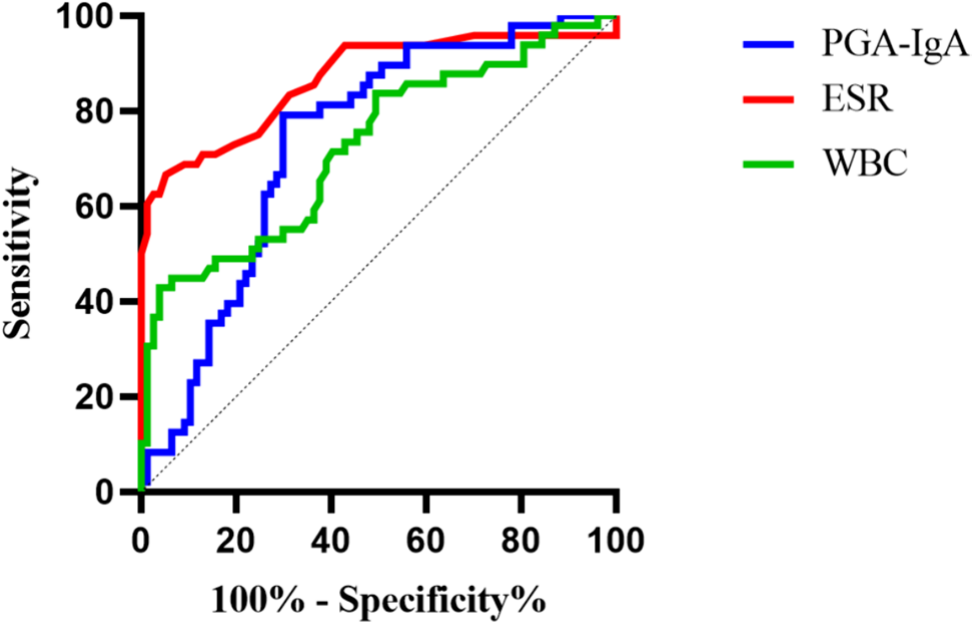
ROC curves of PGA-IgA, CRP, and ESR for identification of disease activity in JIA

We conducted a correlation analysis between the clinical data and PGA-IgA levels (Table 4). PGA-IgA levels were positively correlated with IgA levels, which was understandable. The positive correlation between JADAS27 and sJADAS27 suggested a strong association with disease activity. PGA-IgA was also positively correlated with CRP levels, WBC count, and ESR, indicating a relationship with inflammatory markers, and we found no significant correlation between PGA-IgA levels and age. We further confirmed these associations using a binary regression analysis. As a result, PGA-IgA, WBC, and ESR were still significantly associated with the disease state (Table 5). To compare the ability of PGA-IgA, WBC count, and ESR to judge the degree of disease activity, we drew ROC curves (Fig. 4) and compared their sensitivities and specificities (Table 6). We found that ESR had the largest AUC, and PGA-IgA had the second-largest AUC, with the greatest sensitivity in distinguishing disease stages.

**Table 4:** Correlation analysis of PGA-IgA in JIA with clinical features

Index	Spearman correlation coefficient	*p* value
Age	0.168	0.0608
CRP	0.271	0.0022**
WBC	0.184	0.0398*
ESR	0.293	0.0009***
D-dimer	0.275	0.0533
RF	− 0.019	0.8892
Anti-CCP	− 0.108	0.4391
IgG	0.273	0.0292*
IgA	0.621	< 0.0001****
IgM	0.215	0.088
C3	0.319	0.0110*
C4	0.227	0.0732
Tender joints	0.261	0.0035**
Swelling joints	0.217	0.0155*
JADAS27	0.388	< 0.0001****
sJADAS27	0.610	0.0055**

*CRP* C-reactive protein, *WBC* white blood cells, *ESR* erythrocyte sedimentation rate, *RF* rheumatoid factor, *Anti-CCP* anti-cyclic citrullinated peptide, *IgG* immunoglobulin G, *IgA* immunoglobulin A, *IgM* immunoglobulin M, *C3* complement 3, *C4* Complement 4, *JADAS27* juvenile arthritis disease activity score-27, *sJADAS27* systemic juvenile arthritis disease activity score-27; *p < 0.05, **p < 0.01, ***p < 0.001, ****p < 0.0001.

**Table 5:** Binary logistic regression analysis of the serum inflammatory markers and PGA-IgA in different disease states

Index	RC	*p* value	OR	95% CI
Male	0.306	0.6116	1.358	0.417–4.417
Age	0.043	0.5904	1.044	0.893–1.221
PGA-IgA	1.876	0.0017**	6.527	2.025–21.039
CRP	0.021	0.6849	1.021	0.923–1.129
WBC	0.425	0.0254*	1.529	1.054–2.218
ESR	0.156	0.0014**	1.169	1.062–1.287

*PGA* a-1,4-D-polygalacturonic acid, *IgA* immunoglobulin A, *CRP* C-reactive protein, *WBC* white blood cells, *ESR* erythrocyte sedimentation rate; *p < 0.05; **p < 0.01; ***p < 0.001.

**Table 6:** The values of PGA-IgA, WBC, and ESR in assessing disease activity in JIA patients

Index	AUC	std	*p* value	95% CI for AUC		Cutoff	Sensitivity	Specificity	Youden
				Lower	Upper				
PGA-IgA	0.737	0.045	< 0.0001	0.649	0.824	3.056	0.796	0.701	0.497
ESR	0.869	0.036	< 0.0001	0.798	0.940	15.500	0.673	0.948	0.622
WBC	0.725	0.047	< 0.0001	0.632	0.818	9.225	0.429	0.961	0.390

*PGA* a-1,4-D-polygalacturonic acid, *IgA* immunoglobulin A, *WBC* white blood cells, *ESR* erythrocyte sedimentation rate.

## Discussion

PGA is the primary component of pectin and is widely used in the food, pharmaceutical, and cosmetic industries. Pectin is naturally found in numerous plant fruits, roots, and leaves. In the food industry, pectin assumes a pivotal role as a food additive or ingredient, contributing primarily to gelation, thickening, emulsification, and enhancing stability [6, 7, 11]. Pectin is a high-molecular-weight polysaccharide that is capable of gel formation and partial methyl esterification under specific conditions. This versatile substance, renowned for its hydrophilic groups, possesses robust water-binding properties and finds extensive applications in the food, pharmaceutical, and cosmetic industries. PGA is the primary component of pectin and may play a role in the pathogenesis of autoimmune diseases.

PGA serves as a marker for the recognition of natural IgM antibodies in juvenile serum [12]. The spectrum of natural antibodies is well established to be influenced by both genetic and environmental factors [12–14]. The source of the generated antibodies may encompass PGA found in microorganisms, food, breast milk, or antigenic elements containing the PGA structural domain, as well as autoantigens. A previous study that focused on the correlation between PGA and Henoch-Schönlein purpura in children reported significant increases in PGA-IgG and IgA levels in the Henoch-Schönlein purpura patient group [15]. Dai’s research had indicated a substantial elevation in PGA-IgG and IgA levels among patients with RA but not ankylosing spondylitis (AS) and psoriatic arthritis (PSA), suggesting a close link between circulating anti-PGA antibodies (PGA-Abs) and RA pathogenesis [8]. Our current data further reinforce these findings, demonstrating a significant increase in PGA-IgA levels among patients with pJIA, oJIA, sJIA, and ERA. The classification of JIA in children was different from that in adults. According to pathogenesis and classification criteria, pJIA was most similar to RA, while ERA was most similar to AS. But why were PGA-IgA levels elevated in children with ERA, while they were not elevated in adults with AS? In Dai’s study, when rabbits were immunized subcutaneously with PGA-Abs, they developed severe arthropathies. Similarly, direct injection of affinity-purified PGA-Abs into the synovial cavity resulted in the production of proinflammatory cytokines and local inflammation [8]. Children with ERA often had a higher proportion of peripheral joint involvement compared to patients with AS. Therefore, we speculated that PGA-Abs were more associated with synovial lesions and local inflammatory responses. In our study, PGA-IgG levels were significantly elevated only in children with pJIA, not occurring in other subtypes of JIA. This result also seemed to confirm this point, as Dai’s study similarly observed a significantly greater difference in PGA-IgA than PGA-IgG levels [8]. Further investigation was warranted to elucidate the precise role of PGA-IgA in various inflammatory diseases and subtypes of JIA. The ROC curve presented in this study showed the potential utility of PGA-IgA as a serological diagnostic marker for JIA. Due to the low positivity rates of RF or ACPA in JIA, combining PGA-IgA level detection could help improve the clinical diagnostic rate of JIA.

Furthermore, our study revealed a correlation between PGA-IgA and JIA levels. Initially, the serum levels of PGA-IgA in the active state of patients with JIA were significantly higher than those in the inactive state. Subsequently, PGA-IgA positively correlated with inflammatory indicators, including CRP, ESR, and WBC count. ROC curve analysis showed that PGA-IgA ranked as the second-largest AUC after ESR in the active stage of JIA. Animal experiments have confirmed that antibodies targeting various joint components, such as type II collagen, can induce destructive joint inflammation [16–20]. One potential mechanism by which PGA-specific antibodies (PGA-Abs) may exert arthritogenic effects in vivo involves the direct induction of proinflammatory cytokine production by synovial cells and/or innate immune cells in local tissues [8]. Our study suggested that significantly elevated PGA-IgA levels may contribute to autoimmune inflammation in JIA joints. More importantly, our study showed a significant correlation between PGA-IgA and JADAS27, the most commonly used disease activity score index for JIA. PGA-IgA demonstrated good sensitivity and specificity and could be a complementary biomarker for JIA disease activity. The inflammatory status of the disease would be better assessed when CRP and ESR are normal in some patients with active JIA. Currently, no evidence suggests that pectin consumption increases the risk of arthritis. Conversely, numerous studies have reported the potential of various pectin to ameliorate the symptoms of ulcerative colitis (UC) [21–23]. Furthermore, one study provided evidence for the effectiveness of oral PGA in immune regulation within the lamina propria and gut microbiota. Thus, no significant concern arises regarding the development of arthritis owing to excessive pectin consumption [24].

Although our study represents the first prospective investigation of the relationship between the PGA and JIA, several limitations merit consideration. First, the quantitative measurement of specific serum antibodies in ELISAs is not feasible because of the unavailability of affinity-purified and lyophilized PGA-specific IgG and IgA from human sera. The results were based on optical density (OD) values. Furthermore, the study population was relatively small, encompassing only two centers. This limited sample size may restrict the generalizability of the findings and their ability to detect smaller effects. We cannot exclude the possibility that the observed elevation is an epiphenomenon, given that similar increases are noted in infections [15]. To enhance the robustness of the experimental outcomes, incorporating inflammatory disorders as controls in subsequent experiments would augment the credibility of the findings. Consequently, further studies are required to validate the results obtained in this study.

## Conclusions

In conclusion, elevated levels of anti-PGA antibodies, particularly PGA-IgA, are significantly associated with JIA and may offer valuable diagnostic insights. PGA-IgA demonstrates potential as a sensitive biomarker for assessing disease activity in JIA patients, aiding in the determination of disease activity.

## Data Availability

The original data presented in the study are included in this article. Further inquiries can be directed to the corresponding author.
